# Performance of Biochip system in detecting drug resistant and multidrug-resistant tuberculosis using sputum collected from multiple clinical settings in Zhejiang, China

**DOI:** 10.1038/s41598-018-28955-0

**Published:** 2018-07-12

**Authors:** Kaijin Xu, Shuting Wang, Jie Wu, Zhengwei Liu, Zhongkang Ji, Lin Zheng, Xiuyuan Jin, Jingjing Ren, Shigui Yang, Zhaoxia Li, Jing Yuan, Yanlin Zhao, Lanjuan Li

**Affiliations:** 1 State Key Laboratory for Diagnosis and Treatment of Infectious Diseases, Collaborative Innovation Center for Diagnosis and Treatment of Infectious Diseases, The First Affiliated Hospital, College of Medicine, Zhejiang University, Hangzhou, 310003 China; 2 Zhejiang Provincial Center for Disease Control and Prevention, Hangzhou, 310051 China; 3 Department of infectious Diseases, Dongyang Municipal People’sHospital, Zhejiang, 322100 China; 4 The Third People’s Hospital of Shenzhen, Shenzhen, 518112 China; 5 National Tuberculosis Reference Laboratory of China CDC, Beijing, 102206 China

## Abstract

The objective of the present study was to conduct a multicentre, prospective evaluation of the diagnostic performance of the Biochip system for the detection of drug-resistant tuberculosis using smear-positive sputum specimens. This prospective study evaluated the diagnostic performance of this new platform for drug resistant and multidrug-resistant tuberculosis (MDR-TB) using 1491 smear-positive sputum specimens collected from multiple clinical settings. Using conventional culture-based culturing and drug-susceptibility testing as reference standards, the biochip system had a sensitivity of 86.08% and a specificity of 97.7% for rifampicin (RIF) detection, in detecting isoniazid (INH) resistance, it had a sensitivity of 79.36% and a specificity of 98.71%. With respect to MDR-TB detection, the sensitivity was 78.01% and the specificity was 98.86%. The performance only varies among different sites for RIF resistance, and there are no other statistically difference in diagnostic performance for other variables considered. The Biochip system shows favourable sensitivity and specificity for RIF and INH resistance, along with MDR-TB detection, directly using clinical smear-positive sputum samples. It is an alternative to conventional drug-susceptibility testing (DST) for detecting drug resistance or MDR-TB and is a method worth expanding to clinical settings in China.

## Introduction

In 2013, the World Health Organization (WHO) estimated that mycobacterium tuberculosis (MTB) caused 9 million new active infections and 1.5 million deaths worldwide^1^. China has the third-highest burden of new tuberculosis cases, after India and Indonesia. The emergence of drug-resistant tuberculosis is a major public health concern and may delay global progress towards reaching the WHO post-2015 new End TB Strategy goal of tuberculosis elimination^1^. Rifampicin (RIF) and isoniazid (INH) are the most important first-line anti-tuberculosis drugs, and resistance to these drugs often results in treatment failures and fatal clinical outcomes. Moreover, globally, 4% of new tuberculosis cases and 21% of previously treated cases have multidrug-resistant tuberculosis (MDR-TB), defined as resistance to both INH and RIF; more than half of these patients are located in India, China, and the Russian Federation^1,2^. In a nationwide survey across China in 2007,estimates of MDR-TB prevalence were 5.7% and 25.6% among new and previously treated tuberculosis cases, respectively, and approximately 8% of MDR-TB patients had extensively drug-resistant (XDR) tuberculosis^3^. MDR-TB in China is largely under-detected because culturing and drug-susceptibility testing (DST) are not performed routinely at the local tuberculosis clinics^3^. For example, in 2015, WHO estimated 57,000 cases of MDR-TB while only 5,691 patients actually received laboratory confirmation and effective therapy in China, which means that a substantial proportion of MDR-TB patients have not been detected in China^4^.

Bacterial culture-based DST is still the standard practice for detecting MDR-TB in China. However, culture-based DST, due to slow growth in cultures, contributes to reported treatment initiation delays of 8–80 days from the patient’s first contact with health services^5,6^. The failure to quickly recognize and treat infected patients leads to increased mortality, secondary resistance (including extensively drug-resistant tuberculosis), and ongoing transmission^7^. Additionally, all culture-based conventional methods require a biosafety category III laboratory facility and extensive training of personnel, requirements that are largely unattainable in resource-limited regions^8^. To respond to the urgent need for simple and rapid diagnostic tools in addressing drug resistance and MDR-TB testing, genotypic assays, such as the Cepheid Xpert MTB/RIF(Cepheid, Sunnyvale, CA, USA) and Genotype MTBDR (Hain Lifescience, Nehren, Germany) assays, have been developed^9,10^. These assays usually identify mycobacterial species and mutations (i.e.,*rpoB* and *katG* gene mutations) conferring drug resistance independent of culture; therefore, the time required to obtain results has been reduced to just several hours^11,12^.

**Figure 1:**
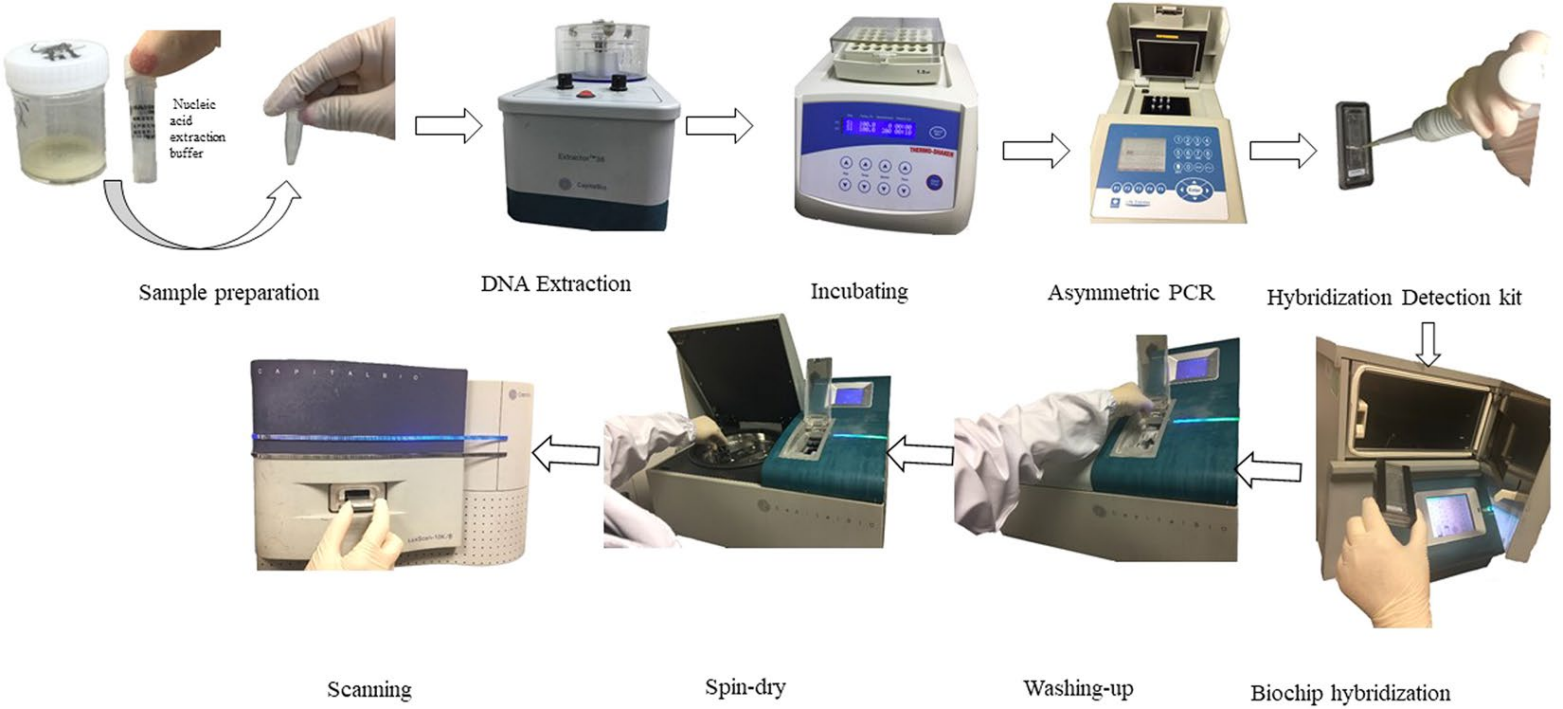
Recommended workflow of the Biochip system The DNA extracted from clinical isolates or specimens is used as a template for PCR. Because the primers are fluorescently tagged, the amplified PCR products also become fluorescently tagged. The PCR products are then hybridized to a microarray slide spotted with mutant gene test sequences, using hybridization buffer. After washing and drying, the chips are imaged using the microarray scanner. The kit interpretation software then analyzes the scan data to generate the test reports, based on the distribution of positive fluorescent probe signals on the microarray.

The Biochip system (Fig. 1), developed by CapitalBio Corporation to detect resistance to RIF and INH through identifying the common mutations in rpoB, katG, and, the inhA gene^13^, shows high efficiency in detecting drug-resistant tuberculosis with notable sensitivity and specificity^13–16^. Previous studies reported that the system had an accuracy of 91.8% in RIF susceptibility prediction and 70.2% in INH, compared with phenotypic DST^16^. However, previous evaluations were mainly carried out on *M. tuberculosis* cultures, few studies have evaluated the performance of the Biochip system with unprocessed sputum samples collected in multiple clinical settings. The objective of the present study was to conduct a multicentre, prospective evaluation of the diagnostic performance of the Biochip system for the detection of drug-resistant tuberculosis in smear-positive unprocessed sputum samples compared against a conventional liquid culture-based referenced standard method in a clinical population.

## Methods

### Study populations

Our prospective diagnostic assessment was done at 8 research sites (hospitals) from 7 cities in Zhejiang Province, China. All tuberculosis patients were referred to the tuberculosis department of each hospital for diagnosis and treatment. Smear-positive sputum samples were confirmed by the Ziehl-Neelsen method and were collected consecutively from the tuberculosis clinics of each hospital between May 15, 2014, and Sept 15, 2015.

### Specimen collection, storage and transportation

Prior to specimen collection, eligible study participants signed a consent form and provided basic demographic data and clinical information concerning the previous history of tuberculosis that was checked and obtained from a request form. Adults with symptoms suggestive of pulmonary tuberculosis or multidrug-resistant tuberculosis were asked to provide a 5-ml sputum sample in a sterile screw cap universal disposable container provided for routine diagnosis. The sputum samples were kept in a refrigerator at a maximum temperature of +4 °C until the specimens were sent for further testing.

### Ethics

The study protocol was reviewed and approved by institutional review boards or ethics committee at The First Affiliated Hospital, College of Medicine, Zhejiang University. The study was conducted in accordance with the protocol. Written informed consent was obtained from all patients. We confirmed that study participation did not alter the standard of care.

### Laboratory methods

Direct smears from each sputum specimen were identified by Ziehl-Neelsen staining for acid fast bacilli (AFB) (National guidelines for tuberculosis laboratories, China Center for Disease Control and Prevention). All sputum specimens were digested and decontaminated with Nacetyl-Lcysteine and sodium hydroxide (NALC-NaOH) for 15 minutes, and the mixture was divided into two identical parts, one for culture and the other for biochip test(Section A, supplementary).

The reagent-specimen mixture was diluted with a PBS buffer to10 mL and centrifuged at 3000 g for 15 min. The supernatant was discarded completely, and the sediment was re-suspended in 0.5 mL of the PBS buffer. The suspension was inoculated on a BACTEC MGIT tube (BD Microbiology Systems, USA) for culture and drug sensitivity tests.

The remaining mixture was examined with the *M. tuberculosis* Drug Resistance Detection Array Kit (Capital Bio, Beijing, China)^13^. The kit contained 5 mutant points for INH resistance, including katG (G315A, G315C, G315T, and C315) and inhA (C-15T). Mutant points for RIF resistance were detected as follows: rpoB/C531G, C531T, CG531AC, A526C, A526G, A526T, C526A, C526G, C526T, T533C, A516G, A516T, G516T, T511C, T511G, C513A, A513T, and C522T.One millilitre of the mixture was centrifuged for 5 min at 10 000 × g to pellet the bacteria. After the supernatant was decanted, the pellet was re-suspended in 1 ml of 0.9% (w/v) saline and then centrifuged at 10 000 × g for 5 min. This supernatant was discarded, and the pellet was re-suspended in 50 μl of 10 mM Tris-EDTA buffer and then transferred to an extraction tube. Total DNA was isolated by vortex in an Extractor™ 36 (Capital Bio) using the highest speed for 5 min. We incubated the extraction tubes for 5 min with a temperature at 95° and then centrifuged at 2348 × g for 1 min. The supernatant was used as a template for amplification. The PCR products were sent to a BioMixer II hybridisation oven (Capital Bio) for 2 h at 50 °C for chip hybridisation for specific gene fragments. Microarrays on the slides were analysed using a confocal LuxScan-10K laser scanner (Capital Bio).

The indirect drug-susceptibility testing of *M. Tuberculosis* isolates to RIF and INH was set up following the manufacturer’s recommended procedures for the MGIT 960 SIRT^17^. All laboratory workers received special biosafety operations training. The clinical laboratories have participated in DST proficiency tests and Biochip tests of the National tuberculosis Reference Laboratory.

### Statistical analysis

Sensitivity and specificity were calculated to evaluate the performance of the Biochip system assay compared to the DST method. Data analysis was performed using R version 3.2.0. The McNemar χ^2^ test or Fisher’s exact test was used to test for significant differences between the variables considered. The difference was judged to be significant if the *P* value was less than 0.05.

### Data Availability

The datasets generated during and/or analysed during the current study are available from the corresponding author on reasonable request.

## Results

### Samples

**Figure 2:**
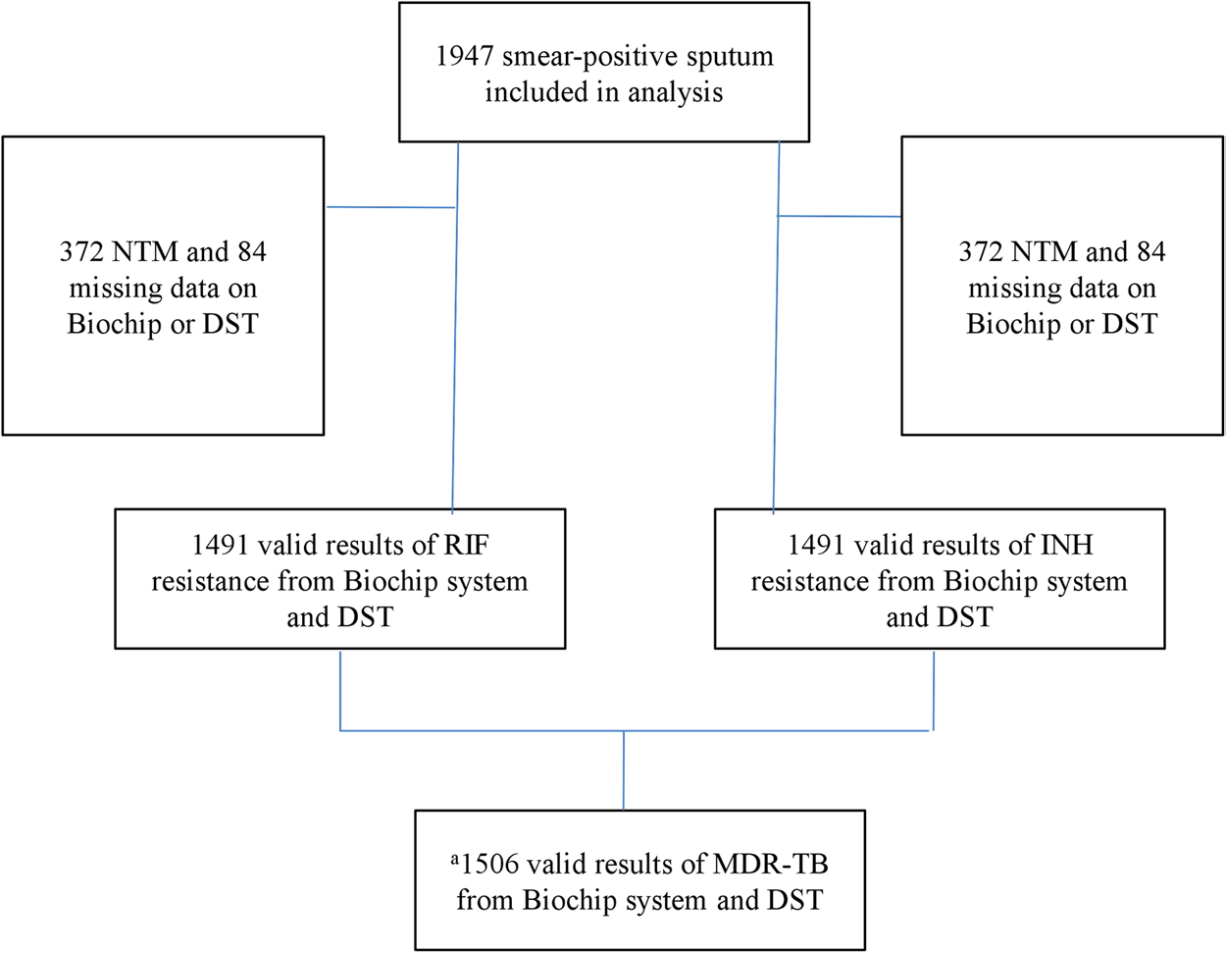
Flowchart of smear-positive sputum included in the analysis. NTM: Nontuberculous mycobacteria, DST: drug-susceptibility testing, RIF: rifampicin, INH: isoniazid, MDR-TB: multidrug-resistant tuberculosis. ^a^The number of samples for MDR-TB calculation is greater because MDR-TB susceptible can be inferred even with only one result on either RIF or INH.

A total of 1947 smear-positive sputum were collected and included in our analysis (Fig. 2). As shown in Table 1, 160 (8.22%) samples had a smear grade of scanty; 1015 (52.13%) had a smear grade of 1+; 376 (19.31%) were 2+ for smear grade; 223 (11.45%) were 3+; and 173 (8.89%) were 4+. Among 1947 smear-positive samples, 1134 (85.33%) were collected from patients who were treatment naïve. The majority of samples were morning sputum (n = 1322, 70.02%), followed by spot sputum and evening sputum. As indicated in Table 1, those 1947 samples were collected from 8 different health institutions in 7 cities. The calculation of the sensitivity and specificity requires all samples to be tuberculous mycobacteria. Therefore, we excluded those nontuberculous mycobacteria (NTM) sputum samples. There were 372 NTM, including 25 mycobacterium abscessus, 36 mycobacterium avium complex, 222 mycobacterium intracellulare, 50 mycobacterium kansasii, and 39 mycobacterium marinum. An additional 84 samples were excluded because of missing results with DST or the Biochip system(Supplementary section B e Table 1). Finally, there were 1491 samples were eligible for RIF and INH sensitivity and specificity calculation (Fig. 2).

**Table 1:** Characteristics of included samples.

Characteristics	N	%
Smear test		
Scanty	160	8.22%
1+	1015	52.13%
2+	376	19.31%
3+	223	11.45%
4+	173	8.89%
Patients		
Treatment naïve	1134	85.33%
Having been treated	195	14.67%
Sample type		
Spot sputum	480	25.42%
Morning sputum	1322	70.02%
Evening sputum	86	4.56%
Site		
Hangzhou	905	46.48%
Jiaxing	178	9.14%
Lishui	106	5.44%
Lingbo	249	12.79%
Wenzhou	192	9.86%
Taizhou	126	6.47%
Jinhua	191	9.81%

The agreement between the Biochip system and DST in detecting drug resistance is provided in e Table 2 section B Supplementary. As shown in Table 2, the system had a sensitivity of 86.08% and a specificity of 97.7% in detecting RIF resistance. The gene mutation points of RIF detected by the Biochip system is presented in e Table 3 section B Supplementary. The performance of the Biochip system does not vary with regard to the smear grade, patients’ treatment history, and type of sputum. The sensitivity of the Biochip system in detecting RIF only differs based on study site. Of special note, the sensitivity of the Biochip system in detecting RIF in Jiaxing is as low as 17.87%, which is substantially lower than the sensitivities in other study sites. The specificity of the Biochip system in detecting RIF does not differ with any variables considered. Our results indicated that the Biochip system had a sensitivity of 79.36% and a specificity of 98.71% in detecting INH resistance. The gene mutation points of INH detected by the Biochip system is presented in e Table 4 section B Supplementary. The performance of the Biochip system with respective to INH resistance detection does not differ with any variables considered. The median time for reporting resistance using the DST method was 11 days, while the median time for reporting resistance using the Biochip system was less than 6 hours.

**Table 2:** Diagnostic performance of the Biochip system for the detection of drug resistance comparing to DST.

	Sensitivity for Rifampin		p value	Specificity for Rifampin		p value	Sensitivity for Isoniazid		p value	Specificity for Isoniazid		p value
Overall	86.08%	(272/316)		97.70%	(1148/1175)		79.36%	(323/407)		98.71%	(1070/1084)	
Smear test			0.9481			0.9997			0.9948			0.998
Scanty	72.00%	(18/25)		95.65%	(66/69)		73.33%	(22/30)		96.88%	(62/64)	
1+	90.97%	(141/155)		98.24%	(614/625)		81.50%	(163/200)		99.31%	(575/579)	
2+	83.12%	(64/77)		96.58%	(226/234)		79.57%	(74/93)		98.62%	(215/218)	
3+	78.79%	(26/33)		98.54%	(135/137)		77.27%	(34/44)		98.43%	(125/127)	
4+	88.46%	(23/26)		97.27%	(107/110)		75.00%	(30/40)		96.88%	(93/96)	
Patients			1			0.8768			1			1
Treatment naïve	92.76%	(141/152)		97.51%	(626/642)		78.97%	(169/214)		98.45%	(572/581)	
Having been treated	93.02%	(40/43)		94.12%	(96/102)		80.00%	(36/45)		98.00%	(98/100)	
Type of sample			0.9886			0.9924			0.9886			0.9924
Spot sputum	80.23%	(69/86)		99.30%	(284/286)		80.23%	(69/86)		99.30%	(284/286)	
Morning sputum	79.54%	(241/303)		98.57%	(757/768)		79.54%	(241/303)		98.57%	(757/768)	
Evening sputum	84.62%	(11/13)		96.00%	(24/25)		84.62%	(11/13)		96.00%	(24/25)	
Site			**0.0462** ^**a**^			0.9721			0.1122			0.9986
Hangzhou	84.48%	(98/116)		98.01%	(492/502)		75.88%	(129/170)		98.86%	(434/439)	
Jiaxing	17.86%	(5/28)		100.00%	(111/111)		21.74%	(5/23)		86.83%	(113/115)	
Jinhua	94.74%	(18/19)		98.65%	(146/148)		100.00%	(21/24)		97.95%	(143/143)	
Lingbo	91.89%	(68/74)		98.26%	(169/172		98.92%	(92/101)		93.55%	(144/145)	
Lishui	95.00%	(19/20)		95.71%	(67/70)		100.00%	(13/13)		100.00%	(77/77)	
Taizhou	100.00%	(20/20)		98.41%	(62/63)		92.00%	(17/17)		100.00%	(64/66)	
Wenzhou	89.80%	(44/49)		92.66%	(101/109)		77.97%	(46/59)		95.96%	(95/99)	

^a^p value is less than 0.05.

DST: drug-susceptibility testing.

In our study, we defined MDR-TB as being resistant to both INH and RIF, and drug susceptible as being susceptible to either INH or RIF or both, which means that we can infer MDR-TB susceptible as long as we know being susceptible to either RIF or INH. Finally, a total of 1506 samples were eligible for MDR-TB calculation. Table 3 presents the results about the performance of the Biochip system in detecting MDR-TB. The Biochip system yields a sensitivity of 78.01% and a specificity of 98.86% in detecting MDR-TB. Our study did not observe significant differences of sensitivity on smear grade, patients’ treatment history, and the type of sputum sample. We also did not observe significant differences of specificity on smear grade, patients’ treatment history, type of sputum sample, and study site.

**Table 3:** Diagnostic performance of the Biochip system for the detection of multidrug-resistant tuberculosis (MDR-TB) comparing to DST.

	Sensitivity for MDR-TB		p value	Specificity for MDR-TB		p value
Overall	78.01%	(220/282)		98.86%	(1210/1224)	
Smear test			0.9476			0.9976
Scanty	62.50%	(15/24)		98.65%	(73/74)	
1+	81.43%	(114/140)		99.07%	(638/644)	
2+	80.30%	(53/66)		98.38%	(243/247)	
3+	75.86%	(22/29)		100.00%	(143/143)	
4+	69.57%	(16/23)		98.28%	(114/116)	
Patients			0.9798			0.9695
Treatment naïve	80.45%	(107/133)		98.81%	((665/673)	
Having been treated	76.92%	(30/39)		97.17%	(103/106)	
Type of sample			0.9775			0.9959
Spot sputum	81.25%	(52/64)		99.35%	(308/310)	
Morning sputum	78.20%	(165/211)		98.85%	(858/868)	
Evening sputum	60.00%	(3/5)		97.30%	(36/37)	
Site			0.0903			1
Hangzhou	76.53%	(75/98)		99.29%	(518/522)	
Jiaxing	18.18%	(4/22)		100.00%	(120/120)	
Jinhua	87.50%	(14/16)		100.00%	(151/151)	
Lingbo	90.28%	(65/72)		98.28%	(171/174)	
Lishui	100.00%	(12/12)		98.72%	(77/78)	
Taizhou	100.00%	(16/16)		100.00%	(67/67)	
Wenzhou	73.91%	(34/46)		94.64%	(106/112)	

MDR-TB: multidrug-resistant tuberculosis; DST: drug-susceptibility testing.

## Discussion

This prospective study evaluated the Biochip system performance on smear-positive sputum specimens from patients with suspected drug-resistant tuberculosis in multiple clinical settings in Zhejiang, China. The Biochip detected drug resistance and MDR-TB with notable sensitivity and specificity, and in a shorter time than the reference DST methods. The Biochip system has been proven to be suitable for application with smear-positive sputum specimens in clinical settings.

This study indicates that the Biochip system has a good overall performance. Our study showed that the sensitivity was around 80% and the specificity was as high as 98% in detecting drug resistant TB. Several commercial diagnostic tools for the detection of drug-resistant tuberculosis have been evaluated in laboratories of various types in China. The sensitivities of these molecular assays for detection of RIF resistance have varied between 87.10% for Xpert MTB/RIF^18^ and 88.3% for Genotype MTBDR^19^, which are in line with the sensitivity of 86.08% in this study. Both Xpert MTB/RIF assay and Hain Line probe assay have been endorsed by the World Health Organization (WHO) as promising new rapid diagnostic technologies with the potential for a large-scale roll-out^20^. Findings from our study suggest that the Biochip system could be applied in a clinical laboratory when a reliable sensitivity assay is required for tuberculosis resistance diagnosis or for screening purposes.

Despite encouraging progress in tuberculosis control, the low detection rate of MDR-TB is one of the major challenges in controlling tuberculosis in China. An accurate and rapid diagnostic method urgently is needed in China. Over the past few decades, a number of new diagnostic technologies have been developed to reduce the time required for detecting MDR-TB. The Biochip (CapitalBio, Beijing, China) is based on molecular analysis from multiple PCRs and reverse hybridization. The Biochip test is designed to identify common mutations for RIF and INH resistance in the rpoB, katG, and the inhA gene. By uncovering mutations in these genes, the Biochip system can detect TB and its multidrug-resistant form in *M. tuberculosis* isolates^16^. The total process is semiautomatic, and the automatic software analysis for the diagnosis of drug resistance eliminates some elements of operator error.

The Biochip system clearly has several advantages over conventional DST. Of greatest clinical relevance is that the Biochip system can deliver resistance results within 6 hours of specimen collection^15^, which is notable because early treatment initiation can be crucial in stopping primary transmission of MDR-TB and reducing mortality^21^.In addition, the Biochip system is based on testing nucleic acid of M. tuberculosisand not a large quantity of live M. tuberculosis, which means there are fewer biosafety concerns compared to conventional DST. Furthermore, the high throughput of the Biochip system reduces the operation time. In our study, findings suggest that the performance of the Biochip system was similar across different study sites. These advantages combined make the Biochip system a more effective, rapid, safe, and cost-effective alternative to conventional DST for RIF and INH resistance and MDR-TB detection. It is a method worth expanding to clinical settings in China. However, it is cautious that our study shows that in very rare case that the sensitivity could be very low (Jiaxing), which we speculate that it is sputum collection and procession issue that cause the low sensitivity. Expanding this method to low-level clinical settings should come with corresponding training on sample collection and procession at low-level clinical settings.

According to a previous study, approximately 95% of TB cases who were RIF-resistant were also resistant to INH^22^. For this reason, many studies used RIF resistance as a proxy to MDR-TB^22,23^. In our study, a smaller proportion (88.23%) of RIF-resistant TB cases that were also resistant to INH has been reported. Although several studies have advocated that detecting resistance to RIF can be used as a marker for MDR-TB with a high accuracy^24^, clearly this practice is not scientifically valid in Zhejiang, China. Xpert MTB/RIF use the RIF resistance as a proxy for MDR-TB. However, the Biochip system directly detects RIF and INH to infer MDR-TB.

## Conclusion

Drug resistance, especially MDR-TB, impede the eradication and the control of tuberculosis. This problem is marked in those country with a heavy tuberculosis burden. The control of infectious disease may require the identification of the infectious agent as the first step. Therefore, the expanded access of rapid, accurate and affordable detection of TB drug resistance or MDR-TB is a prerequisite for TB control. The Biochip system has a favourable performance in detecting TB drug resistance or MDR-TB using convenient clinical sputum samples. More importantly, the whole process is completed within 6 hours. The Biochip system is an alternative to conventional DST for detecting drug resistance or MDR-TB and is a method worth expanding to clinical settings in China.

## Electronic supplementary material

Supplementary
