# Supplementary material for: Performance of Biochip system in detecting drug resistant and multidrug-resistant tuberculosis using sputum collected from multiple clinical settings in Zhejiang, China
Source: Sci Rep. 2018 Jul 12;8:10587. doi: 10.1038/s41598-018-28955-0 (PMC6043617; doi:10.1038/s41598-018-28955-0)
Supplement: Supplementary file 1 — Supplementary [file 41598_2018_28955_MOESM1_ESM.docx]

**Supplementary for “Performance of Biochip system in detecting drug resistant and multidrug-resistant tuberculosis using sputum collected from multiple clinical settings in Zhejiang, China”**

Kaijin Xu^1^ PhD, Shuting Wang^1^ BE, Jie Wu^1^ PhD, Zhengwei Liu^2^ MD, Zhongkang Ji^1^ BE, Lin Zheng^1^ BE, Xiuyuan Jin^1^ MD, Jingjing Ren^1^ PhD, Shigui Yang^1^ MD, Zhaoxia Li^3^ MD, Jing Yuan^4^ MD, Yanlin Zhao^5^ MD, Lanjuan Li^1&^ MD

^&^ Correspondence author

*^1^State Key Laboratory for Diagnosis and Treatment of Infectious Diseases, Collaborative Innovation Center for Diagnosis and Treatment of Infectious Diseases, The First Affiliated Hospital, College of Medicine, Zhejiang University, Hangzhou 310003, China*

*^2^Zhejiang Provincial Center for Disease Control and Prevention, Hangzhou 310051, China*

*^3^Department of infectious Diseases, Dongyang Municipal People’sHospital,Zhejiang 322100, China*

*^4^The Third People’s Hospital of Shenzhen, Shenzhen 518112, China*

*^5^National Tuberculosis Reference Laboratory of China CDC, Beijing 102206, China*

**Correspondence author:**

Prof. Lanjuan Li, MD., *State Key Laboratory for Diagnosis and Treatment of Infectious Diseases, Collaborative Innovation Center for Diagnosis and Treatment of Infectious Diseases, The First Affiliated Hospital, College of Medicine, Zhejiang University, Hangzhou 310003, China.*

Email: [ljli@zju.edu.cn](mailto:ljli@zju.edu.cn)

**Section A: Experimental Flowchart**

The instrument and operation of *M. tuberculosis* Drug Resistance Detection Array Kit (Capital Bio, Beijing, China) and Mycobacteria Identification PCR Diagnostic Kit (Capital Bio, Beijing, China) are similar. They are outlined as follows.

**[Principle]**

Using extracted Mycobacterium tuberculosis DNA as a template, a unique asymmetric PCR technique was applied to amplify target gene fragments. Since the ends of the primers were labelled with fluorescent molecules, all the DNA molecules to be detected during the amplification process were amplified as DNA fragments with fluorescent molecules. The PCR amplification product labelled with a fluorescent molecule and the probe on the chip (layout shown in eFig. 1, 2) were subjected to a hybridization reaction under certain conditions. According to the principle of complementary base pairing, the sequence-matched PCR amplification product and the probe could form a stable secondary structure. Based on the specific arrangement and position of the probe on the chip, relevant information about the tested DNA could be inferred. All oligonucleotide probes and primers listed in e Table 1 and e Table 2 were obtained from Invitrogen (Beijing, China).


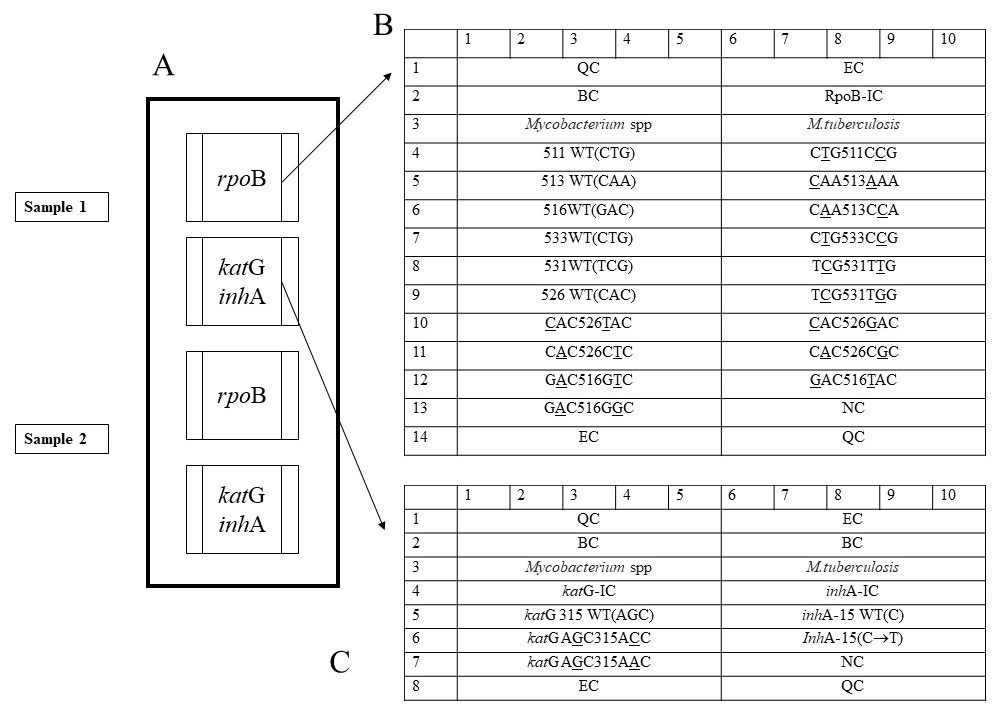


e Figure 1. Sketch map of the chip, array layout of the *rpo*B, *kat*G and *inh*A genes. There were 4 microarrays on each chip, which were used to detect the genotype information for the drug resistance genes in 2 samples, as shown in A. One sample was tested with 2 microarrays. Of these, the rifampin-associated *rpo*B gene was detected in one microarray, and the isoniazid-related *kat*G gene and *inh*A gene promoter were detected in the other microarray. Using the micro-spotting technology for the gene chip, the specific probe for detecting the above genes and various control probes were fixed on the aldehyde-modified glass substrate, with 5 duplicated spots for each of the detection and control probes. The arrangement of the probes is shown in C and B. QC = surface chemistry control; EC = hybridisation control; NC = negative control; IC = mycobacteria and PCR control;


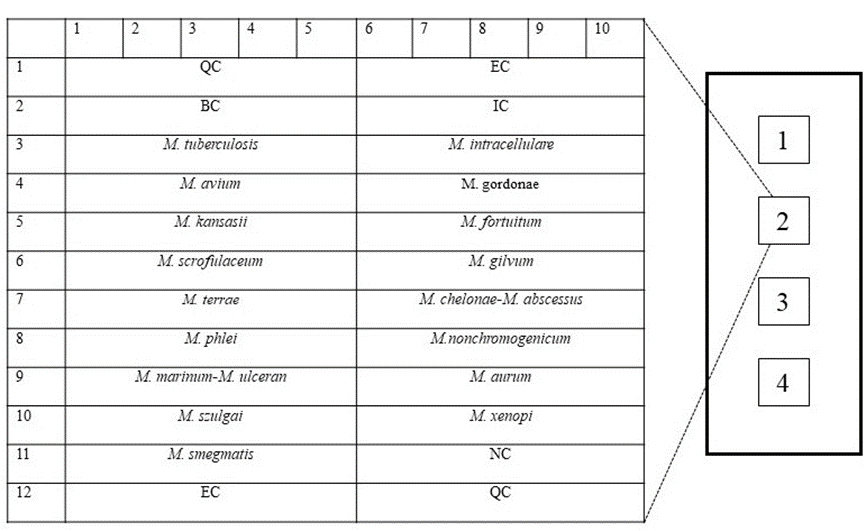


e Figure 2. Sketch map of the chip, array layout of Oligonucleotide probes for the 17 mycobacteria Using the micro-spotting technology for the gene chip, specific probes for detecting the above genes and various control probes were fixed on the substrate, with 5 duplicated spots for each of the detection and control probes, forming a microarray of 12 rows×10 columns. The arrangement of the probes is shown in Fig 2. There were 4 identical microarrays on each chip, and each microarray could detect one sample. QC = surface chemistry control; EC = hybridisation control; NC = negative control; IC = mycobacteria and PCR control;

**e Table 1. Oligonucleotide probes and primers used in this study and the probe threshold and detection limits of the biochip assay for the 17 mycobacteria**

| Oligonucleotide probe and primer | Sequence (5'-3') ^#^ |
| --- | --- |
| BC | DMSO |
| QC | NH_2_-(T)_25_-GCAAGACAAGTGGAAGTGTG-HEX |
| EC | NH_2_-(T)_25_-GCAACCACCACCGGAGG |
| NC | NH_2_-(T)_25_-CCTCTCTCGGACTAATCGCC |
| Mycobacterium spp. | NH_2_-(T)_25_-GCGGGCTCATCCCACAC |
| *M. tuberculosis* | NH_2_-(T)_25_-ACAAGACATGCATCCCGT |
| *M. intracellulare* | NH_2_-(T)_25_-TAAAGACATGCGCCTAAA |
| *M. avium* | NH_2_-(T)_25_-GACATGCGTCTTGAGGTC |
| M. gordonae | NH_2_-(T)_25_-CTTGTGTCCTGTGGTCCT |
| *M. kansasii* | NH_2_-(T)_25_-TTATGCCGGTGTGCAG |
| *M. fortuitum* | NH_2_-(T)_25_-ATGAAGCGCGTGGTCATA |
| *M. scrofulaceum* | NH_2_-(T)_25_-CAACCCACAAAGTGAGCC |
| *M. gilvum* | NH_2_-(T)_25_-CACACACCATGAAGCATG |
| *M. terrae* | NH_2_-(T)_25_-CAGAACATGCATCCCA |
| *M. chelonae-M. abscessus* | NH_2_-(T)_25_-TGGACCACTCACCATGAAGTGTGTG |
| *M. phlei* | NH_2_-(T)_25_-TCCCAGCCATGCAACCAG |
| *M.nonchromogenicum* | NH_2_-(T)_25_-CACACCATGCAGCATG |
| *M. marinum-M. ulcerans* | NH_2_-(T)_25_-CAGAGGACATGAATCCCGT |
| *M. aurum* | NH_2_-(T)_25_-GACATGCATCGCGTAG |
| *M. szulgai* | NH_2_-(T)_25_-CATACGCCTCGGGGTCCT |
| *M. xenopi* | NH_2_-(T)_25_-CCTCCGGTGGTGGTTGC |
| *M. smegmatis* | NH_2_-(T)_25_-CGACCAGCAGGGTGTATT |
| 16S UT prime | TAMRA-TCACTTGCTTCCGTTGAGGTGGCTCAGGACGAACG (*Tm*, 66.2; 48.0°C; 0.4 umol/liter) |
| 16S SS primer | AGCCGTGAGATTTCACGAACA (*Tm*, 50.9°C; 0.2 umol/liter) |

# Underlined letters indicate true point mutations, while lowercase letters indicate the nucleotides introduced to form artificial mismatches. Tm and final concentration of primers are also listed. Tm was calculated using the Primer Premier version 5; QC = surface chemistry control; EC = hybridisation control; NC = negative control; IC = mycobacteria and PCR control; HEX = hexachloro-6-carboxyfluorescein; T25 = 25 consecutive thymidines; DMSO = dimethyl sulfoxide; UT primers=universal unrelated sequence-tagged primers; TAMRA=6-carboxytetramethylrhodamine; SS primer = sequence-specific primers; Tm =melting temp.

**e Table 2 Oligonucleotide probes and primers used in *M. tuberculosis* Drug Resistance Detection Array Kit**

| Drug-resistant gene | Oligonucleotide probe and primer | Sequence (5'-3') # |
| --- | --- | --- |
|  | QC | NH_2_-T_25_-GCAAGACAAGTGGAAGTGTG-HEX |
|  | EC | NH_2_-T_25_-GCAACCACCACCGGAGG |
|  | NC | NH_2_-T_25_-CCTCTCTCGGACTAATCGCC |
| *rpo*B | *rpo*B IC | NH_2_-T_25_-CGGGCACATCCGGCCG |
|  | 531-WT | NH_2_-T_25_-CCCAGCGCCGACAGTC |
|  | 531(G→A) | NH_2_-T_25_-AGCGCCAACAGTCGG |
|  | 533-WT | NH_2_-T_25_-GCCCCAGCGCCGACAGTC |
|  | 533(T→C) | NH_2_-T_25_-GGGCCCCGGCGCCGA |
|  | 526-WT | NH_2_-T_25_-GCGCTTGTGGGTCAA |
|  | 526(C→T) | NH_2_-T_25_-GGCGCTTGTAGGTCAAC |
|  | 526(C→G) | NH_2_-T_25_-CGGCGCTTGTCGGTCA |
|  | 526(A→G) | NH_2_-T_25_-TCGGCGCTTGCGGGT |
|  | 526(A→T) | NH_2_-T_25_-GGGCGCTTGAGGGTC |
|  | 526(C→A) | NH_2_-T_25_-CGGCGCTTGTTGGTCA |
|  | 516-WT | NH_2_-T_25_-TTGTTCTGGTCCATGA |
|  | 516(A→T) | NH_2_-T_25_-GTTCTGGACCATGAA |
|  | 516(A→G) | NH_2_-T_25_-GTTCTGGCCCATGAA |
|  | 511-WT | NH_2_-T_25_-GAATTGGCTCAGCTG |
|  | 511(T→C) | NH_2_-T_25_-ATTGGCTCGGCTGGAT |
|  | 513-WT | NH_2_-T_25_-GAATTGGCTCAGCTG |
|  | 513(C→A) | NH_2_-T_25_-ATGAATTTGCTCAGCT |
|  | 43(A→G) | NH_2_-T_25_-GGCTTCCTCGGAGTG |
|  | 88-WT | NH_2_-T_25_-GTCCTTCACCCGGCC |
|  | 88(A→G) | NH_2_-T_25_-GTCCCTCACCCGGCC |
| *kat*G | *kat*G IC | NH_2_-T_25_-GGCCGGCGCCATGGG |
|  | 315-WT | NH_2_-T_25_-GATGCCGCTGGTGATC |
|  | 315(G→A) | NH_2_-T_25_-GATGCCGTTGGTGAT |
|  | 315(G→C) | NH_2_-T_25_-ATGCCGGTGGTGATC |
|  | 315(C→A) | NH_2_-T_25_-ATGCCTCTGGTGATC |
| *inh*A | *Inh*A IC) | NH_2_-T_25_-GTGGCTGTGGCAGTCAC |
|  | -15-WT | NH_2_-T_25_-CTATCGTCTCGCCGC |
|  | -15(C→T) | NH_2_-T_25_-CCTATCATCTCGCCGC |

# QC = surface chemistry control; EC = hybridisation control; NC = negative control; IC = mycobacteria and PCR control; HEX = hexachloro-6-carboxyfluorescein; T25 = 25 consecutive thymidines.

**[Nucleic acid extraction]**

One millilitre of the liquefied solution was transferred into a 1.5-mL centrifuge tube and centrifuged at 10,000×g for 5 minutes; the supernatant was discarded, and the precipitate was shaken with 1 mL normal saline and centrifuged at 10,000×g for 5 minutes. The supernatant was again discarded, and 50 μL of nucleic acid extraction buffer was added and mixed by shaking. The mixed solution was transferred to a nucleic acid extraction tube and shaken for 5 minutes using an Extractor 36 nucleic acid rapid extractor, followed by incubation in a water bath at 95°C for 5 minutes and centrifugation at 2348×g for 1 minute. The extracted DNA was used as a template for PCR amplification.

**[PCR amplification]**

To test a sample, 18 μL of each PCR amplification agent 1, 2, and 3 was added to 3 rows of new PCR amplification tubes to amplify the sample nucleic acid, positive control, and negative control, respectively, with 2 μL of nucleic acid, 2 μL of the positive control, and 2 μL of negative control respectively added to the amplification tubes and correspondingly labelled. The centrifuge tube was placed in a PCR amplification apparatus, and the PCR amplification reaction was performed according to the thermal cycling programme in the following e table 3.

**e Table 3 PCR amplification programme for Mycobacterium tuberculosis species identification**

| Temperature (°C) | 37 | 94 | 94 | 60 | 72 | 94 | 72 | 72 | 4 |
| --- | --- | --- | --- | --- | --- | --- | --- | --- | --- |
| Time (S) | 600 | 600 | 30 | 30 | 40 | 30 | 60 | 420 | — |
| Number of cycles | 1 | 1 | 45 | | | 20 | | 1 | 1 |

**[Chip hybridization]**

Hybridization reaction mixture (containing the PCR product and buffer, as shown in the following e table 4 and e table 5 respectively)

**e Table 4 Hybridization reaction mixture using in *M. tuberculosis* Drug Resistance Detection Array Kit**

| Hybridization reaction mixture R | | Hybridization reaction mixture H | |
| --- | --- | --- | --- |
| Reactant | Volume (μL) | Reactant | Volume (μL) |
| Hybridization reaction buffer | 9 | Hybridization reaction buffer | 9 |
| PCR product 1 | 3 | PCR product 1 | 3 |
| PCR product 2 | 3 | PCR product 3 | 3 |
| Total | 15 | Total | 15 |

Note

The hybridization reaction mixture R was hybridized to a rifampicin microarray (microarray 1 or 3), and the hybridization reaction mixture H was hybridized to an isoniazid microarray (microarray 2 or 4).

PCR product 1, a control product, was hybridized to both microarrays. PCR product 2 is an amplified product of the rpoB gene and corresponds to the rifampin microarray. PCR product 3 is an amplification product of the katG gene and inhA gene promoter and corresponds to the isoniazid microarray.

**e Table 5. Hybridization reaction mixture using in Mycobacteria Identification Array Kit**

| Reactant | Volume (μL) |
| --- | --- |
| Hybridization reaction buffer | 9 |
| PCR product | 6 |
| Total | 15 |

The BioMixer™ II chip hybridizer was run with hybridization conditions of 50°C for 2 hours at 5 rpm. After denaturation of the hybridization reaction mixture, it was immediately immersed in an ice-water mixture and incubated on ice for 3 minutes. After the hybridization reaction, the hybridization cassette was removed horizontally, and the chip was removed and washed. The obtained chip was immediately placed in a slide holder inside a container (e.g., beaker) containing chip washing solution I equilibrated to room temperature (10-30°C) and washed at 80-100 rpm and room temperature for 3 minutes on a constant-temperature shaker. The chip was then washed with washing solution II equilibrated to room temperature on a constant-temperature shaker at 80-100 rpm and room temperature for 3 minutes, followed by centrifugation at 800 rpm for 5 minutes. The dry chip was then ready to scan.

**[Scanning interpretation]**

The LuxScan 10K-B microarray scanner and corresponding software were used to collect the signals and read the results.

1. The scanner and corresponding software were turned on, and the "laser control" button was clicked and warmed up for 10 minutes.

2. Sample-related information such as "sample number" was entered.

3. After the scanner was warmed up, the "Unload" button was selected to place the chip on the bracket, and the scanner door was gently pushed horizontally, followed by clicking on the "Load" button.

4. The chip number was entered by clicking to select the detection area, and "Select Sample" was chosen to obtain the appropriate sample for each microarray. The "Start" button was selected to scan the chip. The results were displayed on the screen and automatically saved.

5. After one chip was scanned, steps 3 and 4 were repeated to scan the next chip until all chips were scanned.

6. Data query and printing were performed through "Data Query".

7. After completing all operations, the laser was turned off, the software exited, and the scanner turned off.

**[Interpretation of the results]**

The signal value of the probe was compared with the reference value. Some results of our study are shown in e Figure 3.

**
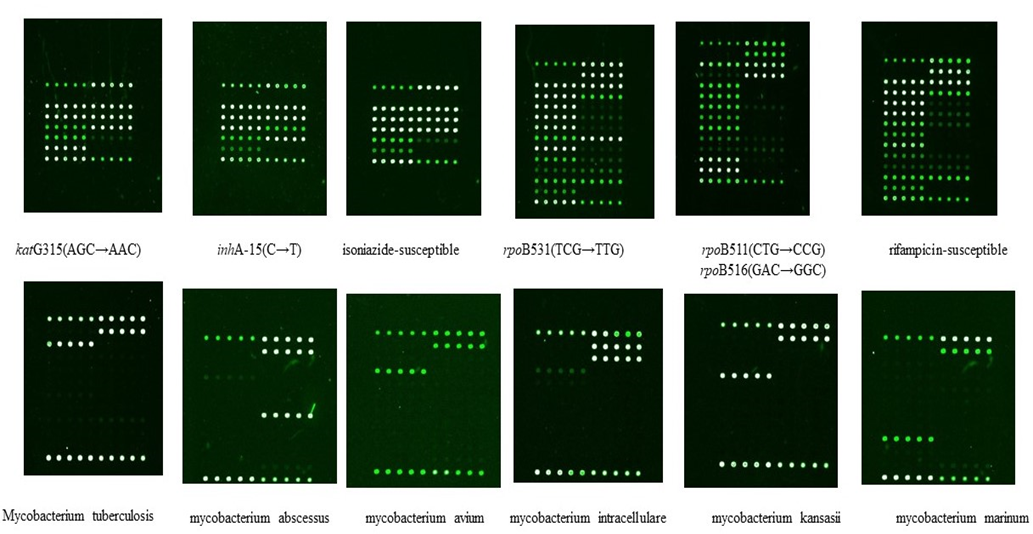
**

e Figure 3. Schematic diagram of the probe arrangement on a microarray chip

| **e Table 1** Samples with invalid results from the Biochip system and DST | | | |
| --- | --- | --- | --- |
| Methods | | Results | Explanations |
| The Biochip system | |  |  |
|  | N=32^*^ | In-determine | In sufficient DNA |
| DST | |  |  |
|  | N=56^*^ | Contamination | Inappropriate operation |

**Section B: additional results**

| *2 samples having missing data on both DST and the Biochip system  DST=drug-susceptibility testing  **eTable2** Agreement of DST and the Biochip system on TB drug-resistance testing | | | | | | |
| --- | --- | --- | --- | --- | --- | --- |
| The Biochip system | | DST | | Overall agreement (95%CI) | Positive agreement  (95%CI) | Negative agreement(95%CI) |
|  |  | Resistant | Susceptible |  |  |  |
| Rifampin | |  |  |  |  |  |
|  | Resistant | 272 | 27 | 95.32 (94.28-96.34) | 88.46 (86.03-89.51) | 97.07 (96.05-98.05) |
|  | Susceptible | 44 | 1175 |  |  |  |
| Isoniazid | |  |  |  |  |  |
|  | Resistant | 323 | 14 | 93.42  (92.23-95.62) | 86.83 (85.62-88.01) | 95.62 (95.41-96.89) |
|  | Susceptible | 84 | 1070 |  |  |  |
| MDR-TB | |  |  |  |  |  |
|  | Resistant | 220 | 14 | 94.45 (93.32-95.46) | 85.27 (84.26-86.31) | 96.96 (95.89-97.92) |
|  | Susceptible | 62 | 1210 |  |  |  |

DST=drug-susceptibility testing; MDR-TB= multidrug-resistant tuberculosis; CI= confidence interval

**;**

**e Table** **3 gene mutations of 252 rifampin -resistant tubercle bacilli,**

| **Mutant points** | **Mutant times for single site, no, (%)** |
| --- | --- |
| inhA-15 (C→T) | 47(18.7) |
| *katG*315(AGC→ACC), (AGC→AAC) | 205(81.3) |
| total | 252 |

**e Table 4 gene mutations of 216 rifampicin-resistant tubercle bacilli**

| Mutant points | No of strains，(%) |
| --- | --- |
| Single mutation point |  |
| *roo*B511(CTG→CCG) | 10(4.6) |
| *roo*B513(CAA→AAA) | 1(0.5) |
| *roo*B513(CAA→CCA) | 1(0.5) |
| *roo*B516(GAC→TAC) | 10(4.6) |
| *roo*B516(GAC→GTC) | 8(3.7) |
| *roo*B516(GAC→GGC) | 3(1.4) |
| *roo*B526(CAC→CGC) | 6(2.8) |
| *roo*B526(CAC→CTC) | 2(0.9) |
| *roo*B526(CAC→GAC) | 8(3.7) |
| *roo*B526(CAC→TAC) | 16(7.4) |
| *roo*B531(TCG→TGG) | 3(1.4) |
| *roo*B531(TCG→TTG) | 127(58.8) |
| *roo*B533(CTG→CCG) | 9(4.2) |
| Double mutation points |  |
| *roo*B511(CTG→CCG); *roo*B513(CAA→CCA) | 1(0.5) |
| *roo*B511(CTG→CCG); *roo*B516 (GAC→GGC) | 9(4.2) |
| *roo*B511(CTG→CCG); *roo*B531(TCG→TGG) | 1(0.5) |
| *roo*B516(GAC→GTC); *roo*B531(TCG→TTG) | 1(0.5) |
| Total | 216(100) |
